# Prolapsing Intrigue: A Case of Superior Vena Cava Mass Visualized by Echocardiography From the Subcostal Window Unveiling an Anterior Mediastinal Type B2 Thymoma

**DOI:** 10.7759/cureus.52352

**Published:** 2024-01-16

**Authors:** Hasan Kazma, Mouin Fouani, Mustafa Olleik, Leila Akil, Malek Mohammed

**Affiliations:** 1 Cardiology, Bahman Hospital, Beirut, LBN; 2 Medicine, Faculty of Medical Sciences, Lebanese University, Beirut, LBN; 3 Hematology-Oncology, Bahman Hospital, Beirut, LBN; 4 Pathology, Bahman Hospital, Beirut, LBN; 5 Invasive Cardiac Laboratory, Bahman Hospital, Beirut, LBN

**Keywords:** ct scan of chest, anterior mediastinal mass, right atrial mass, superior vena cava mass, transthoracic 2d echocardiography, echocardiographic windows, point-of-care-ultrasound, thymoma, superior vena cava

## Abstract

Imaging the superior vena cava (SVC) during two-dimensional (2D) transthoracic echocardiographic examination is challenging and should be performed routinely. Here, we present a case where a lower (juxta-atrial) SVC mass was seen prolapsing into the right atrium by 2D transthoracic echocardiography; in this case, the imaging of the lower (juxta-atrial) SVC was done from the subcostal window. It was not possible to image the SVC from the suprasternal, right supraclavicular, left parasternal, or apical windows.

CT scan of the chest with intravenous contrast was done in this case and showed an anterior mediastinal mass invading the SVC and prolapsing into the right atrium. CT-guided biopsy proved the mass to be a type B2 thymoma.

## Introduction

Imaging the superior vena cava (SVC) should be a part of any comprehensive two-dimensional (2D) transthoracic echocardiographic examination [1,2]. In patients with indwelling catheters used for hemodialysis, chemotherapy, or parenteral nutrition and those with pacemaker leads, imaging of the SVC and mainly its lower (juxta-atrial) part is a crucial noninvasive test to look for complications like vegetations and thrombus formation [3,4]. Also in patients with a mediastinal mass invading the SVC, ultrasound imaging of the SVC is imperative during echocardiographic examination [5]. There are specific imaging windows that have to be used to assess the SVC during echocardiographic examination; these windows will allow assessment of the SVC by ultrasound and Doppler (pulsed wave Doppler and color flow Doppler). The recommended windows are the right supraclavicular window, the suprasternal window, the apical window (modified apical five-chamber view), the left parasternal window (modified left parasternal short axis view at the cardiac base), and the subcostal window [6]. 

In our patient, transthoracic echocardiography identified a right atrial mass; the origin or the attachment of this mass could not be depicted from the left parasternal or apical windows and it was the subcostal window that allowed us to confirm that the mass originated from the lower (juxta-atrial) SVC and was prolapsing into the right atrium. We could not visualize the SVC from other windows. An enhanced CT scan of the chest showed a large anterior mediastinal mass invading the SVC and reaching the right atrium. CT-guided biopsy of the mass was done and the pathology showed a type B2 thymoma.

## Case presentation

**Figure 1:**
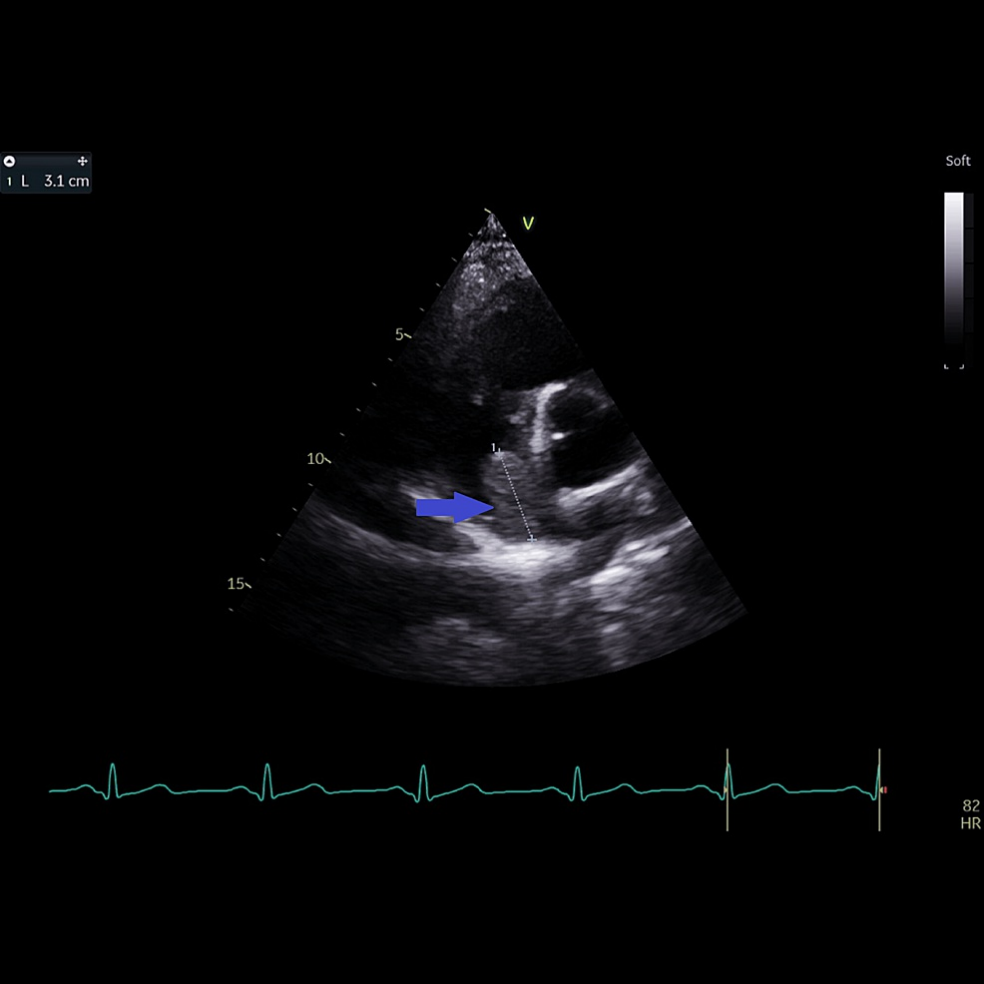
Modified short-axis view at the base of the heart from the left parasternal window showing the right atrial mass (blue arrow); but the origin or the attachment of the mass could not be visualized from this view. The lower (juxta-atrial) superior vena cava could not be visualized from this view.

**Figure 2:**
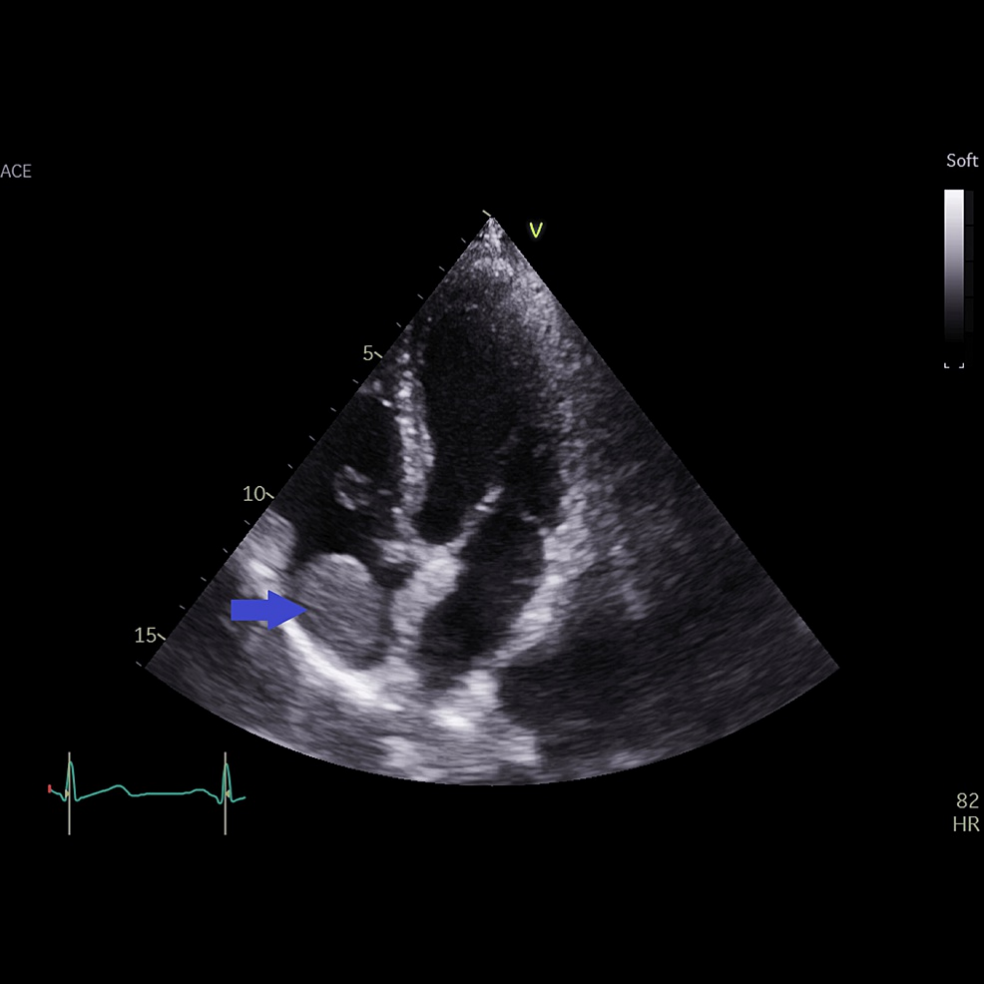
Apical four-chamber view showing the right atrial mass (blue arrow); the origin or the attachment of the mass could not be visualized from this view. The lower (juxta-atrial) superior vena cava could not be visualized from this view.

**Figure 3:**
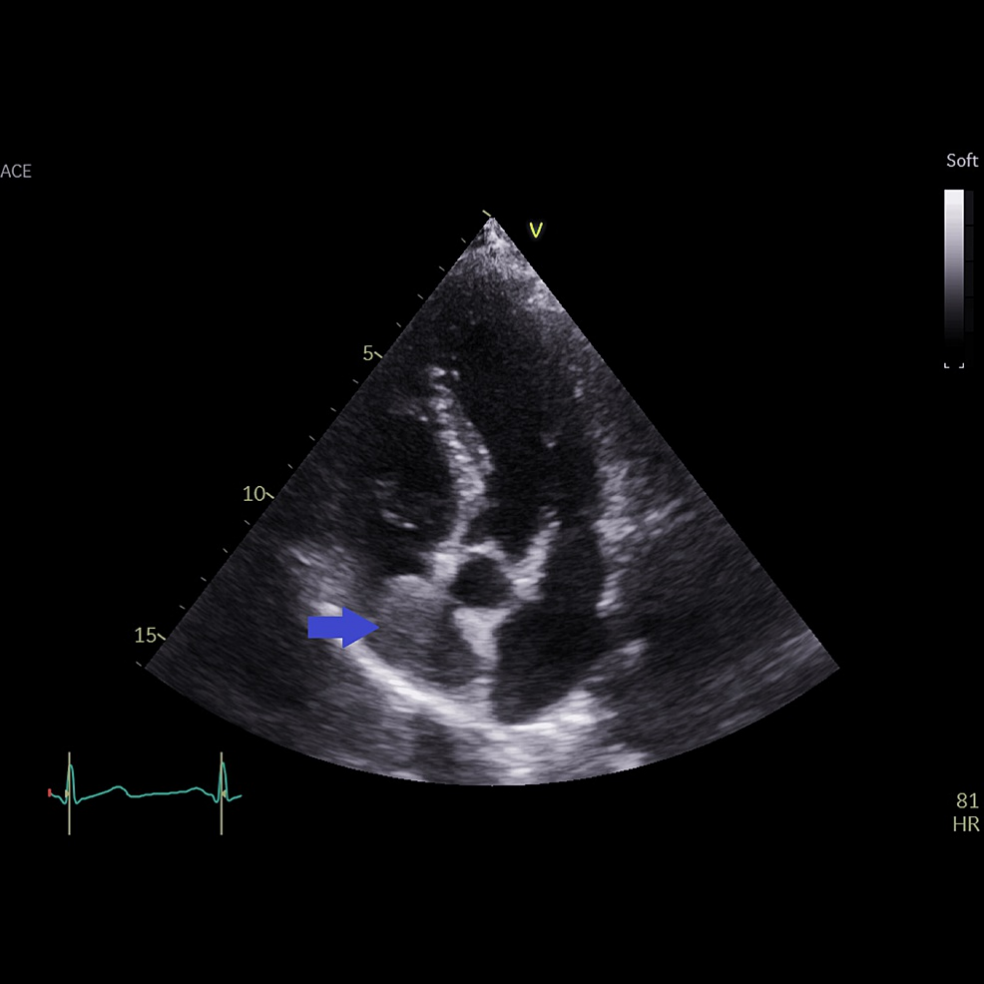
Apical five-chamber view showing the right atrial mass (blue arrow); the origin or the attachment of the mass could not be visualized from this view. The lower (juxta-atrial) superior vena cava could not be visualized from this view.

A 58-year-old male patient, who was a heavy smoker, presented to our hospital because of dyspnea and palpitations. He also reported a history of fascial and neck edema that resolved on its own one week before his presentation. On physical exam, his blood pressure was 120/80, heart rate was 86/minute, and respiratory rate was 16/minute, no fever was noted; he had moderate neck vein distension with normal heart and lung exam, no palpable lymph nodes, no hepato-splenomegaly, and no leg edema. Because of the presence of dyspnea and neck vein distention, 2D transthoracic echocardiography was performed; a mass was noted inside the right atrium from the left parasternal and apical windows, but its attachment or origin could not be visualized (Figures 1-3); the lower (juxta-atrial) SVC could not be visualized from these windows.

**Figure 4:**
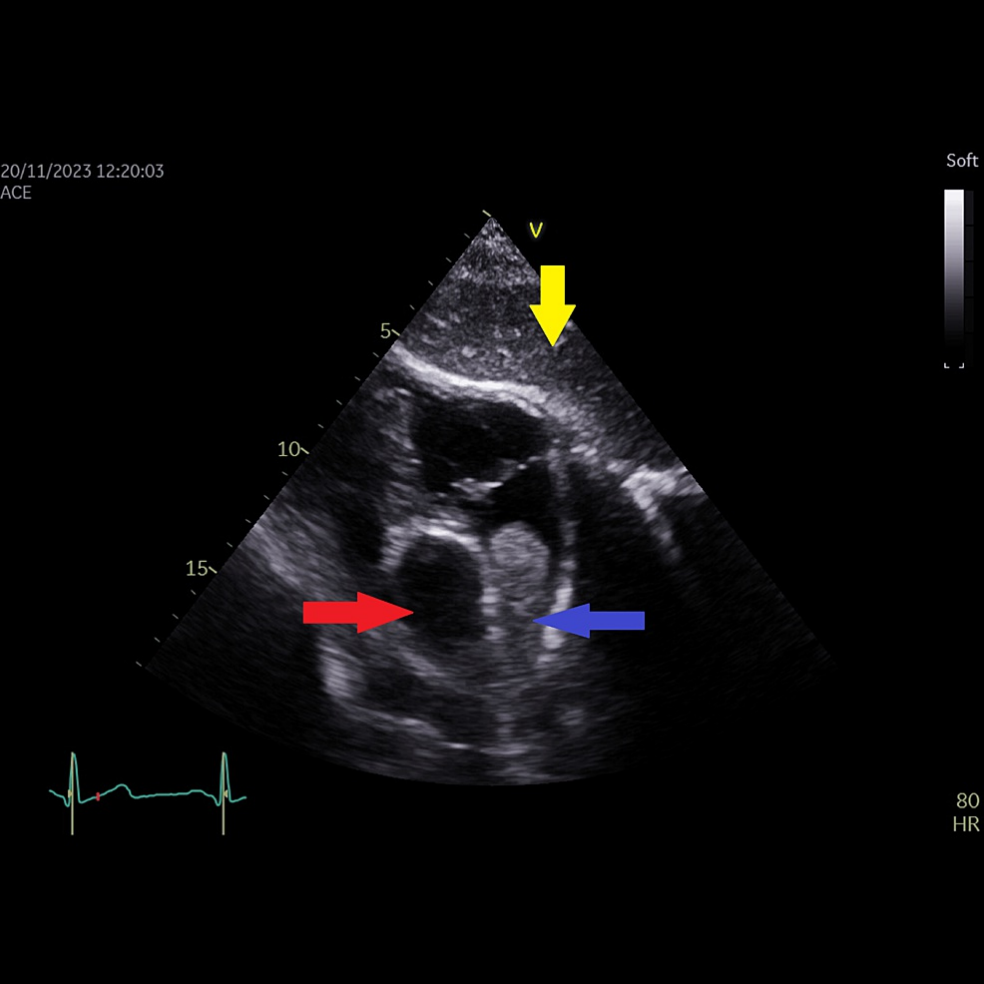
Modified subcostal view of the right atrium showing actually that the right atrial mass originated from the lower (juxta-atrial) superior vena cava and is prolapsing into the right atrium. Blue arrow showing the right atrial mass originating from the lower (juxta-atrial) SVC. Red arrow showing the ascending aorta. Yellow arrow showing the liver.

**Figure 5:**
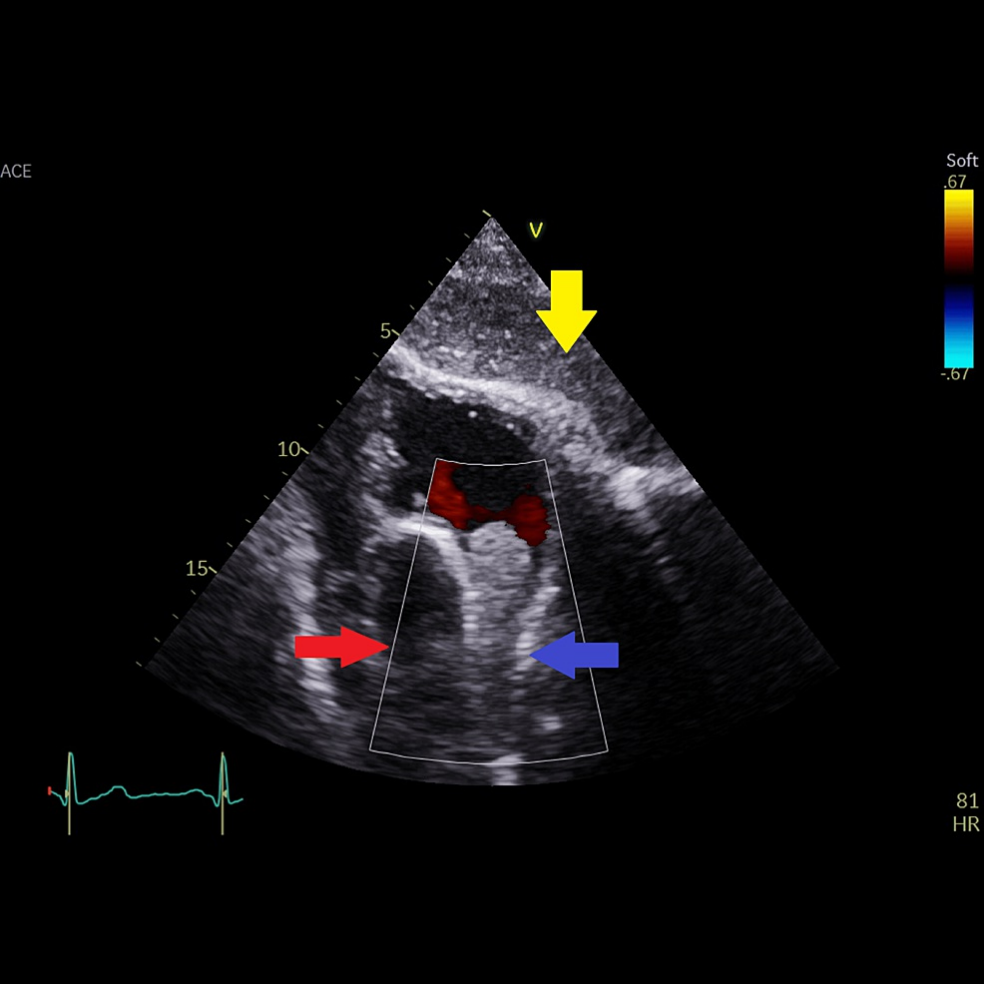
Same modified subcostal view of the right atrium with color coding showing no aliasing of color flow around the right atrial mass originating from the lower (juxta-atrial) superior vena cava, indicating no major obstruction to flow. Blue arrow showing the right atrial mass originating from the lower (juxta-atrial) SVC. Red arrow showing the ascending aorta. Yellow arrow showing the liver.

**Figure 6:**
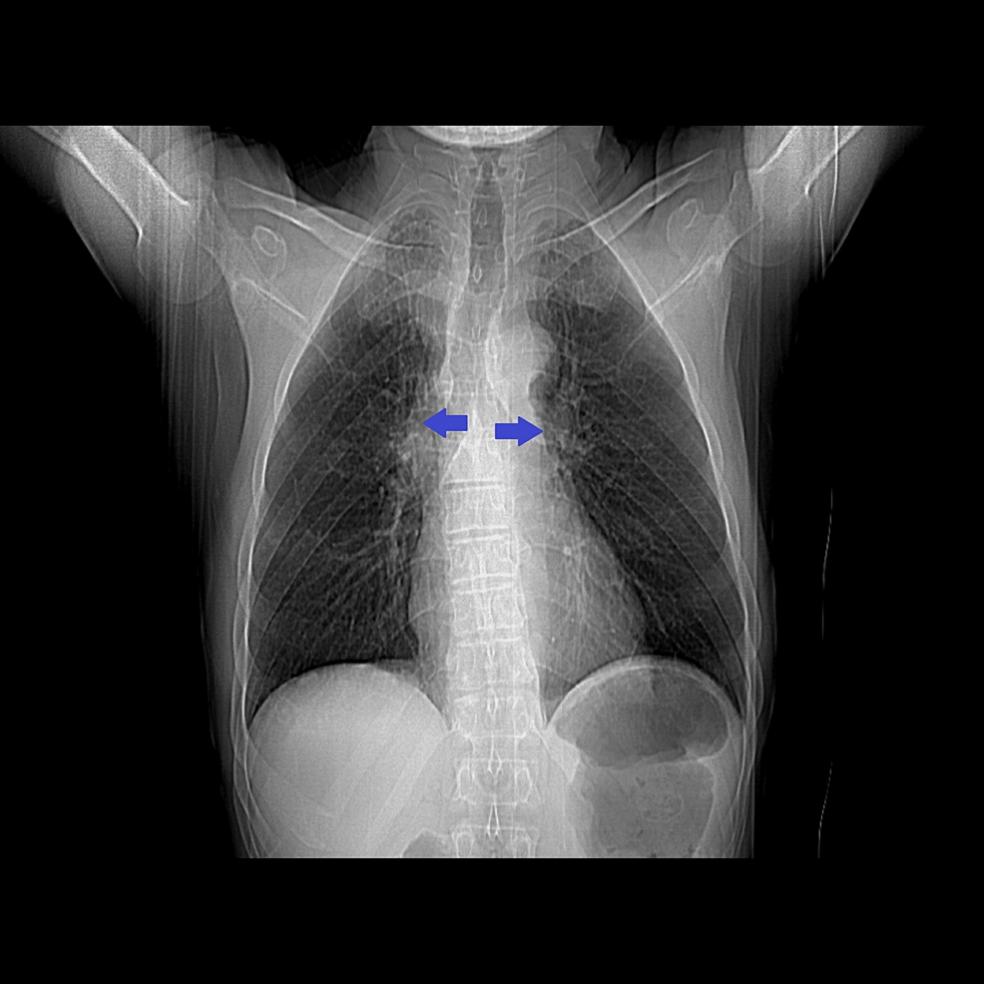
CT scout view of the chest showing an enlargement of the mediastinum (blue arrows).

The subcostal window (modified subcostal view of the right atrium) allowed visualization of the lower (juxta-atrial) SVC and the mass was seen originating from the distal part of the SVC and prolapsing into the right atrium (Figures 4, 5). The upper SVC could not be visualized from the suprasternal or the right supraclavicular windows. CT scout view of the chest showed an enlargement of the mediastinum (Figure 6).

**Figure 7:**
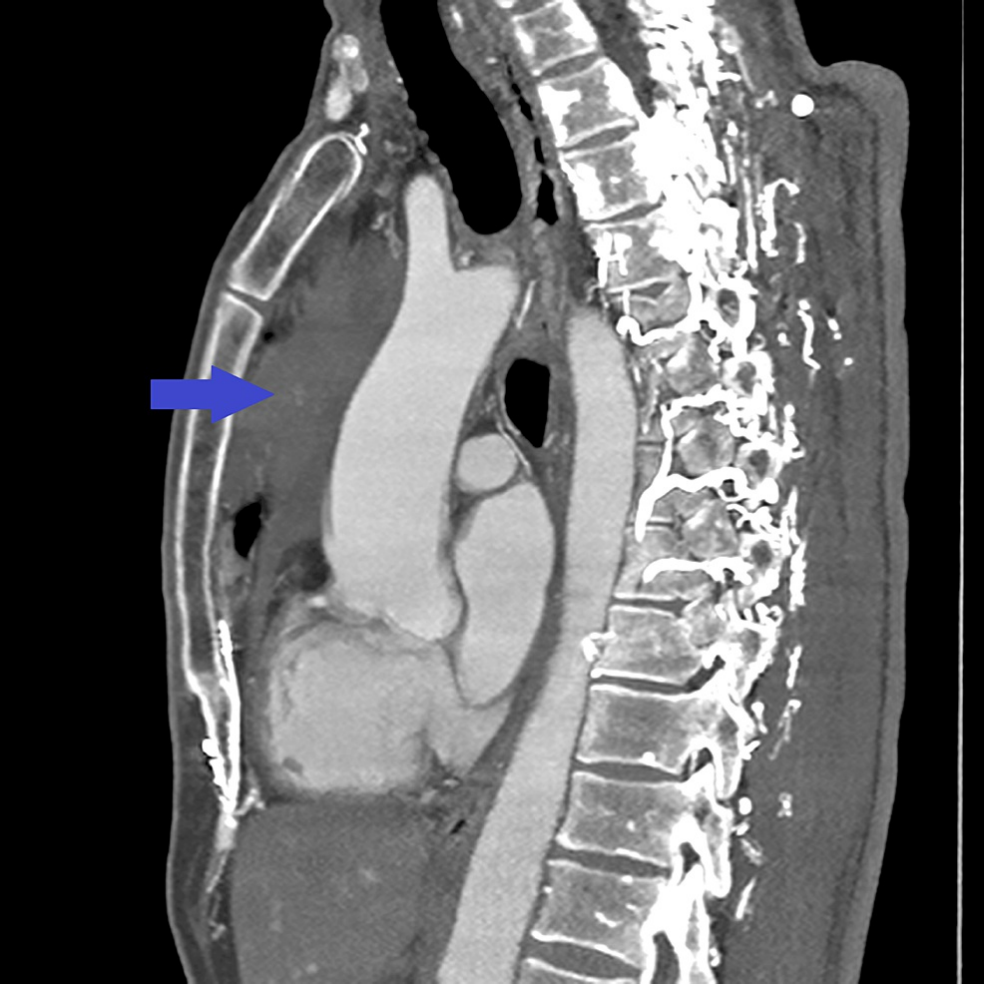
Enhanced CT scan of the chest showing a large anterior mediastinal mass (blue arrow).

**Figure 8:**
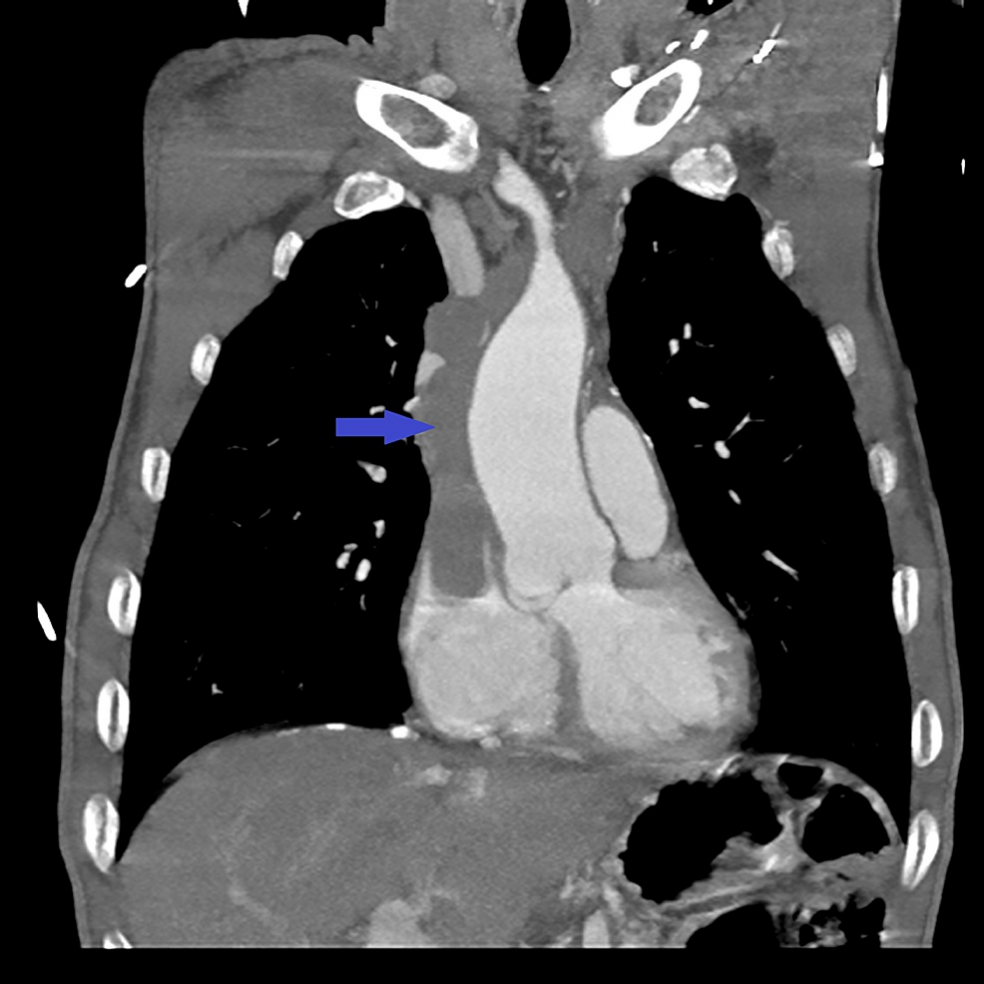
Enhanced CT scan of the chest showing the anterior mediastinal mass invading the superior vena cava and prolapsing into the right atrium (blue arrow).

**Figure 9:**
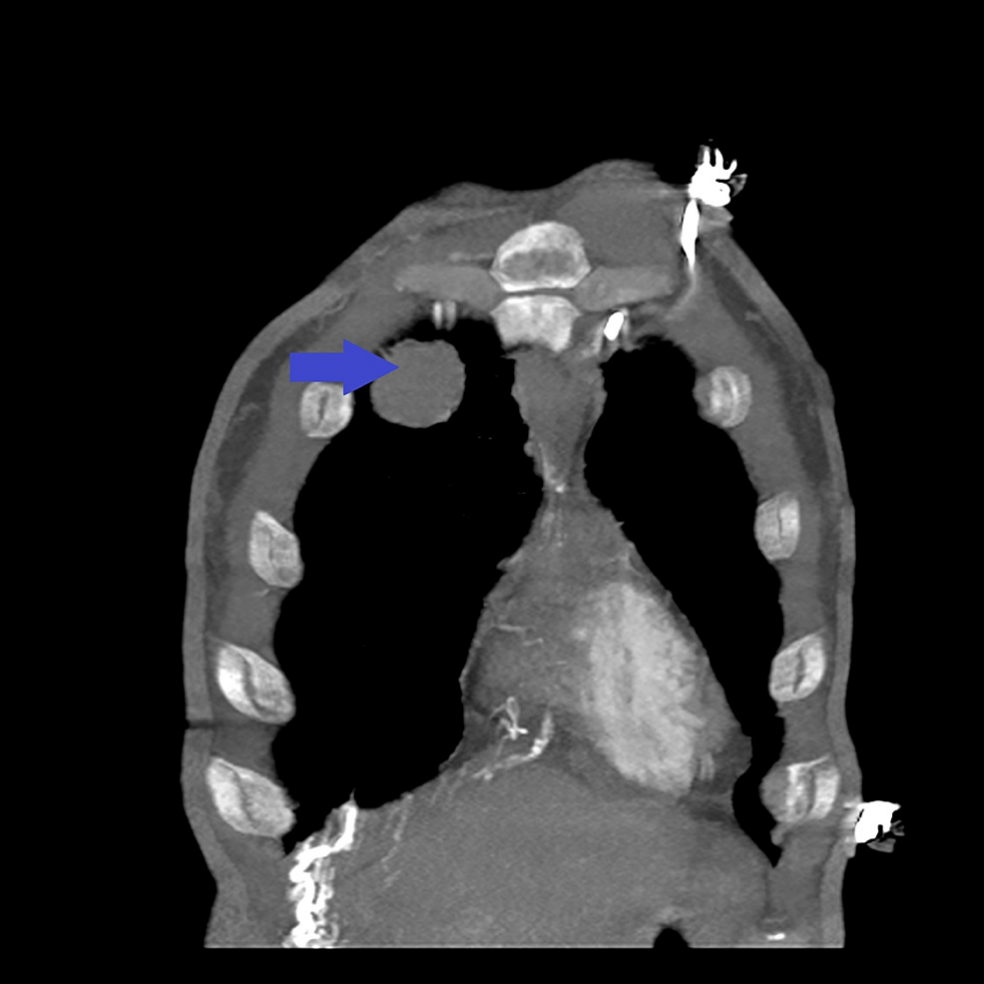
Enhanced CT scan of the chest showing a large metastasis to the right upper lobe (blue arrow).

An enhanced CT scan of the chest was performed which showed a large anterior mediastinal mass invading the SVC and prolapsing into the right atrium (Figures 7, 8). CT scan of the chest also showed metastasis to the right upper lung field (Figure 9).

**Figure 10:**
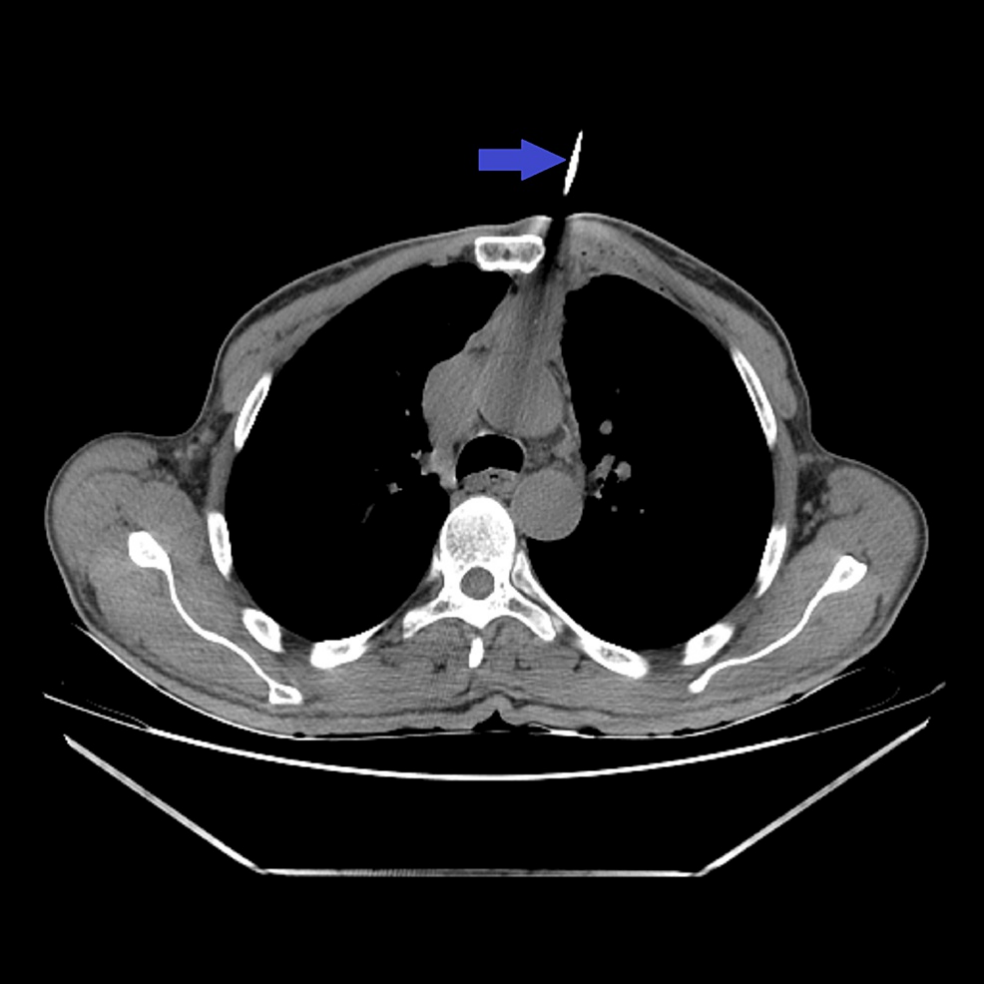
Core needle biopsy being done under CT guidance (blue arrow).

**Figure 11:**
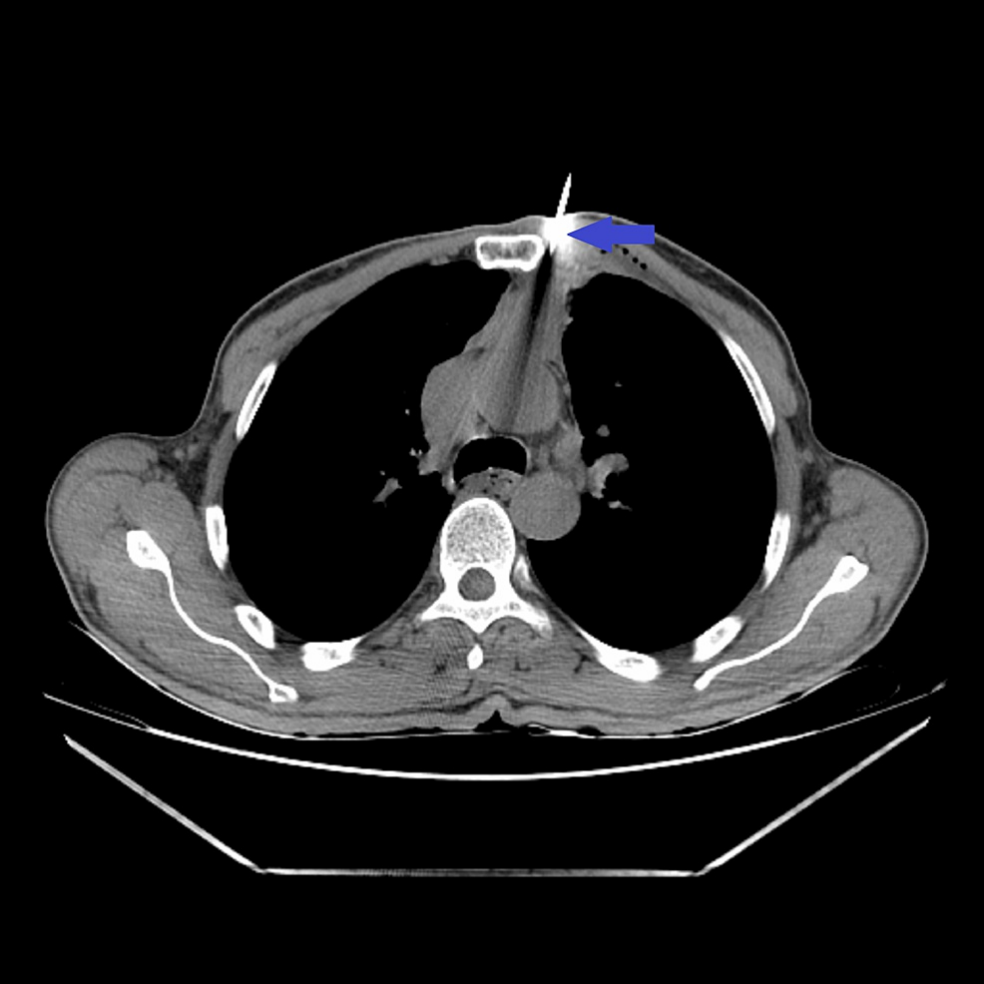
Needle being advanced into the tumor under CT guidance to perform the core biopsy (blue arrow).

A core needle biopsy of the anterior mediastinal mass was done under CT guidance (Figures 10, 11).

**Figure 12:**
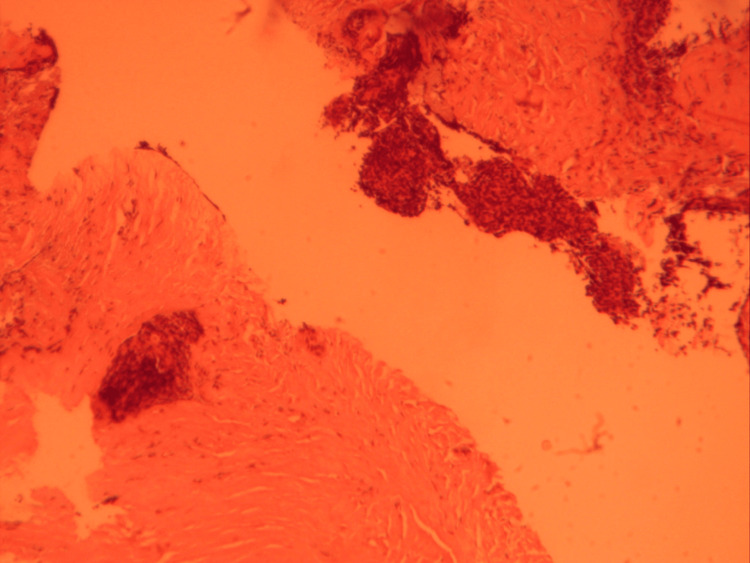
Pathology specimen showing thymoma type B2.

**Figure 13:**
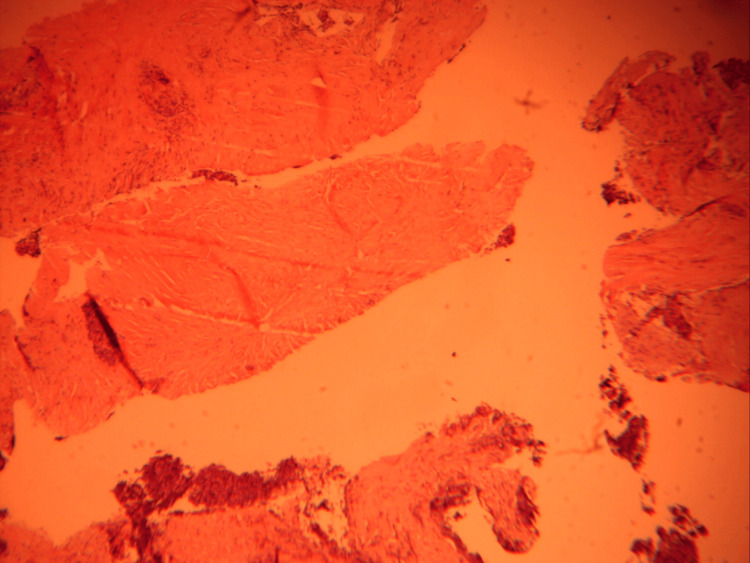
Pathology specimen showing thymoma type B2.

Pathology showed thymoma type B2 (Figures 12, 13). An immunohistochemistry study revealed that the P63 was positive in epithelial cells; the lymphocytes expressed CD2, CD99, and TdT; TTF1 and synaptophysin were negative. Ki67 was highly expressed >60%.

The patient was referred to oncology and cardiothoracic surgery departments for further evaluation and treatment.

## Discussion

The SVC is a straight tube, about 7 cm long, that is formed by the confluence of the right and left innominate veins and ends by entering the right atrium at its superior pole. The SVC descends vertically to the right of the aorta and trachea down from the level of the first costal cartilage to about the level of the third costal cartilage, where it enters the right atrium, approximately posterior to the right sternal border [7].

There are specific windows (views) that have to be used when imaging the upper part of the SVC: the right supraclavicular window and the suprasternal window; the lower (juxta-atrial) SVC can be imaged from the apical window (modified apical five-chamber view), the left parasternal window (modified left parasternal short axis view at the cardiac base) and the subcostal window [1,2]. In the presence of a right atrial mass, it is crucial to assess the origin or the site of attachment of this mass so it is important to image both the lower (juxta-atrial) SVC and the inferior vena cava (IVC) looking for a relation of this right atrial mass to the lower (juxta-atrial) SVC or the IVC. Thus, it is very important to master the technical skills to visualize the lower (juxta-atrial) SVC and IVC during transthoracic echocardiography [6,8].

The suprasternal or right supraclavicular windows have been used for imaging the SVC and recording Doppler flow signals from the SVC [9]. Suprasternal or right supraclavicular scanning with the transducer placed in the fossa between the sternal and clavicular heads of the sternomastoid muscle, while the patient lying supine, reveals the top 4 cm of the SVC and also the right innominate vein, its formation by the right subclavian and internal jugular veins, and often the left innominate vein [2].

The SVC can be visualized through the subcostal approach with the patient lying supine, as a straight narrow tube opening into the right atrium at its upper pole. Imaging of the SVC from two opposite windows (supraclavicular and subcostal) provides complementary information. The lower part of the SVC is better defined from the subcostal view; the upper half of the SVC is more clearly seen from the right supraclavicular or suprasternal views [2].

The SVC can be visualized by the modified apical five-chamber view: the patient is positioned halfway between supine and left lateral decubitus, the transducer placed in the 5th left intercostal space at the anterior axillary line with the index mark pointing to the patient’s right shoulder, by gentle side tilting of the transducer more medially (between − 40 and − 80 degrees), the lower (juxta-atrial) SVC could be visualized and studied [6].

The SVC can be visualized by the modified left parasternal short axis view of great cardiac vessels: the patient is slightly turned to the left, somewhere between supine and left lateral decubitus position, the transducer is put in the third left intercostal space, close to the left sternal border with the index mark pointing to the suprasternal notch, by mild and gentle tilting of the transducer toward the right shoulder (between − 15 and − 30 degrees) the lower (juxta-atrial) SVC could be seen and assessed in this view [6].

In our patient, the IVC was easily identified from the subcostal window and it was not related to the right atrial mass. We could not visualize the SVC from the suprasternal or the right supraclavicular windows possibly because the anterior mediastinal mass was invading it, also we could not visualize the lower (juxta-atrial) SVC from the parasternal or apical windows possibly due to technical difficulties; and since we systematically image the SVC from multiple windows, we insisted on viewing the lower (juxta-atrial) SVC from the modified subcostal window and in fact this window allowed us to identify that the right atrial mass originated from the lower (juxta-atrial) SVC. 

Ultrasound evaluation of the superior vena cava during the 2D transthoracic echocardiographic examination will lead to an increase in diagnostic yield: from central catheter thrombosis to superior vena cava syndrome and invasion of the SVC by a tumor [3-5]; furthermore, the point-of-care ultrasound (POCUS) can allow clinicians, by evaluating the SVC from different windows during transthoracic echocardiography, to make a rapid diagnosis for example of a SVC thrombosis at the bedside in a patient in respiratory distress with SVC syndrome [4].

The confirmation of SVC and right atrial invasion by the anterior mediastinal mass was done with an enhanced CT scan of the chest which is a robust technique due to its high resolution [10], but the interesting fact is that echocardiographic imaging of the lower (juxta-atrial) SVC from the subcostal window did detect the involvement of the IVC by the tumor [6].

Our case report of a large anterior mediastinal type B2 thymoma invading the superior vena cava and protruding into the right atrium is a rare case according to our literature review [5], and what makes this case more unique is that the lower (juxta-atrial) SVC involvement by the Thymoma was diagnosed by 2D transthoracic echocardiography, this case report emphasizes on the importance of imaging the systemic veins routinely during 2D transthoracic echocardiography.

Our patient was referred to the oncology and cardiothoracic surgery departments for further evaluation and therapy; chemotherapy, radiotherapy and large resection of the thymoma with reconstruction of the SVC on cardiopulmonary bypass have been done previously [11,12], and these treatment options were explained to the patient. 

Despite having a type B2 thymoma, our patient did not complain of muscle weakness, in fact, 50% of patients with thymomas have myasthenia gravis while 15% of patients with myasthenia gravis have thymomas [13].

## Conclusions

In this case report, our systematic approach of imaging the SVC and its lower (juxta-atrial) part from different windows, during transthoracic echocardiography allowed us to detect the SVC involvement by the tumor. Thus, it should be a routine practice while performing echocardiography to visualize the SVC from multiple windows. Furthermore, implementation of POCUS may allow clinicians to make very important and sometimes critical diagnosis very safely and accurately at the bedside by imaging the SVC and the IVC.
